# Sedation versus general anesthesia on all-cause mortality in patients undergoing percutaneous procedures: a systematic review and meta-analysis

**DOI:** 10.1186/s12871-024-02505-w

**Published:** 2024-04-02

**Authors:** Xuesen Su, Zixin Zhao, Wenjie Zhang, Yihe Tian, Xin Wang, Xin Yuan, Shouyuan Tian

**Affiliations:** 1 The First College for Clinical Medicine, Shanxi Medical University, No. 56 Xinjian South Road, Taiyuan, Shanxi People’s Republic of China; 2 College of Anesthesia, Shanxi Medical University, No. 56 Xinjian South Road, Taiyuan, Shanxi People’s Republic of China; 3 Department of Anesthesiology, First Hospital of Shanxi Medical University, No. 85 Jiefang South Road, Taiyuan, Shanxi People’s Republic of China; 4 John Muir College, University of California San Diego, 8775 Costa Verde Blvd, San Diego, CA USA; 5 Shanxi Province Cancer Hospital/Shanxi Hospital Affiliated to Cancer Hospital, Chinese Academy of Medical Sciences No. 3, Workers’ New Village, Xinghualing District, Taiyuan, Shanxi People’s Republic of China

**Keywords:** Sedation, General anesthesia, All-cause mortality, Systematic review, Meta-analysis

## Abstract

**Background:**

The comparison between sedation and general anesthesia (GA) in terms of all-cause mortality remains a subject of ongoing debate. The primary objective of our study was to investigate the impact of GA and sedation on all-cause mortality in order to provide clarity on this controversial topic.

**Methods:**

A systematic review and meta-analysis were conducted, incorporating cohort studies and RCTs about postoperative all-cause mortality. Comprehensive searches were performed in the PubMed, EMBASE, and Cochrane Library databases, with the search period extending until February 28, 2023. Two independent reviewers extracted the relevant information, including the number of deaths, survivals, and risk effect values at various time points following surgery, and these data were subsequently pooled and analyzed using a random effects model.

**Results:**

A total of 58 studies were included in the analysis, with a majority focusing on endovascular surgery. The findings of our analysis indicated that, overall, and in most subgroup analyses, sedation exhibited superiority over GA in terms of in-hospital and 30-day mortality. However, no significant difference was observed in subgroup analyses specific to cerebrovascular surgery. About 90-day mortality, the majority of studies centered around cerebrovascular surgery. Although the overall pooled results showed a difference between sedation and GA, no distinction was observed between the pooled ORs and the subgroup analyses based on RCTs and matched cohort studies. For one-year all-cause mortality, all included studies focused on cardiac and macrovascular surgery. No difference was found between the HRs and the results derived from RCTs and matched cohort studies.

**Conclusions:**

The results suggested a potential superiority of sedation over GA, particularly in the context of cardiac and macrovascular surgery, mitigating the risk of in-hospital and 30-day death. However, for the longer postoperative periods, this difference remains uncertain.

**Trial registration:**

PROSPERO CRD42023399151; registered 24 February 2023.

**Supplementary Information:**

The online version contains supplementary material available at 10.1186/s12871-024-02505-w.

## Introduction

Sedation, a type of anesthesia, has seen escalating utilization across various surgical procedures, particularly those involving minimally invasive techniques and catheterization, such as transcatheter aortic valve replacement (TAVR), transcatheter left atrial appendage closure, and endovascular thrombectomy [1–3]. Sedation frequently plays an integral part in enhanced recovery after surgery (ERAS) protocols, designed to minimize the reliance on general anesthesia (GA), especially in critically ill patients. Its implementation has been associated with reduced rates of intensive care unit (ICU) admissions and postoperative cognitive impairment [4]. Conversely, these percutaneous procedures are typically performed under GA, which does not appear to yield worse patient outcomes when compared to sedation according to a previous study [5]. GA provides complete intraoperative analgesia and deep sedation, ensuring a stable surgical environment and enhancing safety. Consequently, the choice between GA and sedation in percutaneous procedures remain a topic of ongoing debate, as multiple patient and procedural factors may influence the decision [6].

Of particular importance to surgical patients, especially those in critical condition, is mortality, rendering it the primary outcome of interest when comparing these two anesthesia techniques. Thus, determining whether GA or sedation can affect postoperative mortality has become a clinical concern. However, the impact of different anesthesia approaches on all-cause mortality in patients undergoing percutaneous procedures has produced inconsistent findings. A retrospective study found that monitored anesthesia care (MAC) was associated with lower 30-day mortality and comparable 3-year mortality in patients undergoing TAVR [7]. In contrast, results from a multicenter randomized controlled trial (RCT) investigating the same surgical procedure indicated that GA reduced 30-day mortality compared to sedation [8].

To further clarify the results of this comparison, several systematic reviews and meta-analyses incorporating numerous clinical studies have been conducted. Hung KC et al. identified that sedation was associated with lower risks of 30-day mortality by pooling data from 24 clinical studies on TAVR [9]. In contrast, a meta-analysis of three RCTs involving patients undergoing intracranial mechanical thrombectomy showed no significant difference in mortality [10]. Although variations in percutaneous procedures may contribute to the observed discrepancies, this remains speculative. Therefore, our comprehension of the relationship between the type of anesthesia used and all-cause mortality in patients undergoing percutaneous procedures remains limited.

To address this gap in knowledge, we conducted a systematic review and meta-analysis focusing on sedation alone or combined with local anesthesia and GA, excluding cases where regional nerve block or intraspinal anesthesia was employed. Specifically, our analysis aimed to investigate the impact of GA and sedation on postoperative all-cause mortality by incorporating a larger sample size.

## Methods

This meta-analysis was conducted according to the guidelines of Preferred Reporting Items for Systematic Reviews and Meta-Analysis (PRISMA) [11] and followed a protocol registered on the international prospective register of systematic reviews (PROSPERO: CRD42023399151).

### Search strategy

Two investigators conducted an independent and comprehensive search of online databases, namely PubMed, EMBASE, and the Cochrane Library, with no language restrictions, to identify articles on the key terms "general anesthesia," "sedation," and "mortality." The search encompassed articles available up until February 28, 2023. In the event of any discrepancies during the literature search process, a third reviewer was consulted to facilitate a thorough discussion and reach a consensus. The specific search terms employed for each electronic database were detailed in Supplemental Table 1.

### Study selection

All references identified through the implemented search strategy were exported to Endnote V.X9 (Thomson Reuters, Philadelphia, USA) and subjected to an independent evaluation by two reviewers. The initial screening involved assessing the titles and abstracts of all retrieved search results, followed by a detailed examination of the full texts of potentially relevant articles. For inclusion in this meta-analysis, studies were required to satisfy the following PICOS criteria: (1) Population: patients undergoing percutaneous procedures with either GA or sedation, (2) Intervention: sedation, encompassing various depths and drug usage, (3) Comparison intervention: GA with tracheal intubation or laryngeal mask ventilation, (4) Outcome: risk estimates of death or mortality at least one postoperative time point, and (5) Study design: RCT and cohort study. Studies that did not meet these criteria, including those with insufficient data, nonhuman studies, abstracts only, and protocols, were excluded. In instances where disputes arose regarding the eligibility of specific papers, a comprehensive discussion was held involving a third reviewer to resolve.

### Quality assessment

The risk of bias in RCTs was independently evaluated by two reviewers using the Cochrane risk of bias criteria [12, 13] and the Newcastle–Ottawa Scale (NOS) was employed for cohort studies [14]. The Cochrane risk of bias criteria encompassed several domains, namely random sequence generation, allocation concealment, blinding of participants and personnel, blinding of outcome assessment, completeness of outcome data, selective reporting, and other biases. Each study was assessed for potential bias, with a rating of "Low," "High," or "Unclear" assigned accordingly. On the other hand, the NOS consisted of eight categories addressing methodological quality, and each study received a score out of a maximum of 9 points. A score ranging from 0 to 6 denoted a low-quality study, while a score of 7 to 9 indicated a high-quality study. The overall certainty of the evidence was evaluated following the Grading of Recommendations Assessment, Development, and Evaluation (GRADE) framework [15–18]. Any disagreements about quality assessment were resolved through a full discussion with a third reviewer.

### Data extraction

Two reviewers independently conducted the extraction of relevant details from the included studies, with any discrepancies in data extraction resolved through thorough discussion involving a third reviewer. The extracted information encompassed the following: (1) Study characteristics, including the first author's name, study design, publication year, country, and sample size; (2) Participant characteristics, such as age, the proportion of males, and the specific surgeries or procedures included; (3) The number of deaths and survival outcomes reported at all time points for both intervention groups; (4) Adjusted risk estimates, accompanied by 95% CIs, for mortality obtained from any statistical models employed in the studies.

### Statistical analysis

The statistical analyses were conducted utilizing Stata software version 17.0 (Stata Corp.). For dichotomous data, the relevant information extracted from each study included the total number of patients in each group and the number of patients experiencing the outcome of death. Risk ratios (RRs) and their corresponding 95% CIs were synthesized to evaluate the outcome. To calculate the log ORs, HRs, and relative risks, we utilized those (along with their respective 95% CIs) derived from the included articles that compared sedation versus GA. Irrespective of whether the data were dichotomous or in the form of risk estimates, the outcomes were pooled using a random-effects model due to the expected clinical and methodological diversity among the included studies.

To explore statistical heterogeneity among the pooled effects, the Cochran Q statistic was employed, and the extent of heterogeneity was quantified using the I^2^ metric (significant heterogeneity defined as I^2^ > 50% and *p* < 0.05) [19]. For outcomes comprising more than 10 studies, the potential risk of publication bias in the included studies was evaluated through visual examination of funnel plots and quantitative Egger's tests.

Subgroup analyses were conducted to investigate potential sources of significant heterogeneity and assess the impact of important factors on all-cause mortality. These factors included: (1) study design, categorized as RCT, matched cohort study, and non-matched cohort study; and (2) type of surgery or procedure, classified as cardiac and macrovascular, cerebrovascular, or other surgeries. Furthermore, sensitivity analysis was performed by systematically excluding individual studies to assess the influence of each study on the overall pooled estimate.

## Results

### Search results and study characteristics

**Fig. 1:**
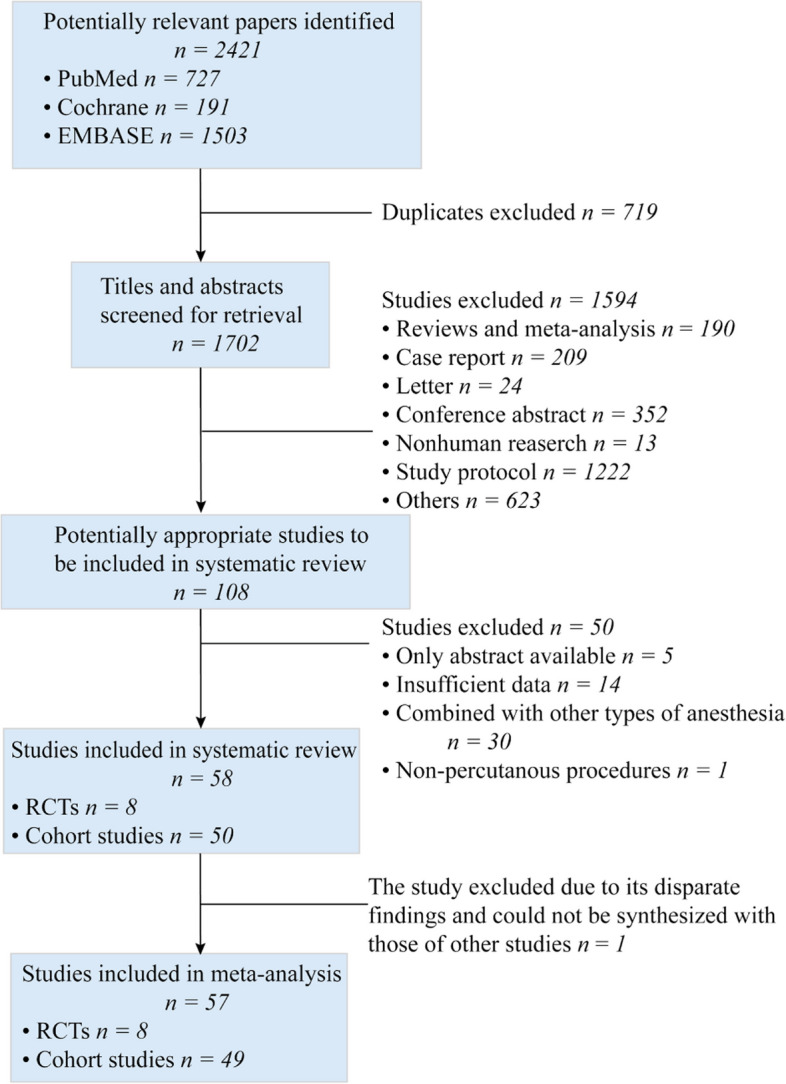
Flow diagram of study selection

The initial search strategy found 2421 articles, and after excluding papers that were duplicates or did not meet the inclusion criteria, 108 full-text articles of potentially relevant studies were identified. Following a full-text review, an additional 50 articles were excluded. Specifically, to more accurately compare GA and sedation in terms of all-cause mortality, we excluded studies on GA or sedation combined with other types of anesthesia including nerve block and epidural anesthesia. Finally, 57 studies were included in our meta-analysis (Fig. 1), including 8 RCTs, and 49 cohort studies (including 12 matched cohort studies).

Table 1 and Supplemental Table 2 presents the study characteristics. A total of 61,945 patients were involved. 29,843 and 32,102 patients completed surgery under GA and sedation, respectively. The mean or median age of the enrolled patients varied from 6.5 to 85.4 years with the proportion of males ranging from 14.3% to 100%. Of the final included studies, 39 involved cardiac and macrovascular surgery, 18 on cerebrovascular surgery, and one on ERCP. Various medications for GA (total intravenous or intravenous-inhalational anesthesia, intubation, or laryngeal mask general anesthesia) or sedation (e.g., conscious sedation, deep sedation, and MAC) were used across the studies, as detailed in Supplemental Table 2*.* The NOS score of the 50 cohort studies ranged from 6 to 9 (Table 1), and the risk of bias assessment for 8 RCTs was shown in Supplemental Fig. 1.

**Table 1:** Study characteristics

Study	Country	Multi-center	Study design	Age (yr)^a^	N	Male (%)^a^	Surgery	NOS scores
Mikus, 2021 [20]	Germany	No	Cohort	6.5 ± 6.0 vs. 7.3 ± 6.2 (months) ^b^	803	100 vs. 100	Cardiac Catheterization	9
Zaouter, 2018 [21]	France	No	Cohort	80.2 ± 7.5 vs. 81.8 ± 8.4^b^	234	48.5 vs. 51.2	TAVI	8
Weyland, 2021 [22]	Switzerland	No	Cohort	76 (64, 81) vs. 75 (66, 82)^c^	105	68.3 vs. 46.7	Endovascular stroke treatment	8
Toppen, 2017 [23]	USA	No	Matched cohort	82.4 ± 11 vs. 83.5 ± 9^b^	196	50.3 vs. 46.9	TAVR	8
Thiele, 2020 [8]	Germany	Yes	RCT	81.4 ± 5.7 vs. 81.8 ± 5.3^b^	437	48.6 vs. 49.1	TAVI	^𝛿^
Theron, 2014 [24]	UK	No	Cohort	64.6 ± 12.9 vs. 68 ± 10.6^b^	183	83.2 vs. 88.2	Implantation of Pacing Device	8
Téllez-Alarcón, 2022 [25]	Brazil	No	Cohort	78.1 (75.8, 80.4) vs. 80.4 (78.9, 81.9)^d^	158	55.6 vs. 48.8	TAVI	8
Stragier, 2019 [26]	Belgium	No	Cohort	81.7 (80.0, 83.5) vs. 81.3 (79.6, 83.1)^d^	178	48.2 vs. 53.8	TAVI	8
Shan, 2018 [27]	China	Yes	Matched cohort	63.5 (54.5, 74.3) vs. 65.5 (59, 72.3)^c^	228	66.7 vs. 61.4	Endovascular thrombectomy	8
Schönenberger, 2016 [28]	Germany	No	RCT	71.8 ± 12.9 vs. 71.2 ± 14.7^b^	150	65.8 vs. 54.5	Endovascular thrombectomy	^𝛿^
Palermo, 2016 [29]	USA	No	Cohort	79.6 ± 0.9 vs. 85.4 ± 9.1^b^	65	76.2 vs. 68.2	TAVI	7
Sammour, 2021 [7]	USA	No	Cohort	80 ± 10.2 vs. 81 ± 9^b^	998	56.1 vs. 41.8	TAVR	9
Renner, 2019 [30]	Germany	No	Cohort	82 ± 6.1 vs. 82 ± 6.4^b^	200	41.1 vs. 51.6	TAVI	8
Ren, 2020 [31]	China	No	RCT	69.21 ± 5.78 vs. 69.19 ± 6.46^b^	90	54.2 vs. 57.1	Endovascular thrombectomy	^𝛿^
Rassaf, 2014 [32]	Germany	No	Cohort	75 ± 8.7 vs. 74.5 ± 8^b^	21	63.6 vs. 90	Percutaneous mitral valve repair	6
Powers, 2019 [33]	USA	Yes	RCT	69.5(59, 78) vs. 70.5(59, 79)^c^	92	54 vs. 48	Endovascular thrombectomy	^𝛿^
Piayda, 2021 [34]	Germany	Yes	Cohort	75 ± 8 vs. 75 ± 9^b^	949	67.1 vs. 62.9	Transcatheter left atrial appendage closure	8
Patzelt, 2017 [35]	Germany	No	Cohort	74 ± 10 vs. 78 ± 8^b^	271	62.5 vs. 43.7	Percutaneous mitral valve repair (PMVR)	7
Pani, 2017 [36]	USA	No	Cohort	83 (78, 89) vs. 83 (77, 88)^c^	97	51 vs. 55	TAVR	8
Musuku, 2021 [37]	USA	No	Matched cohort	81 (74, 86) vs. 83 (76, 88)^c^	296	55 vs. 48	TAVR	9
Mosleh, 2019 [38]	USA	No	Matched cohort	80.31 ± 9.31 vs. 82.06 ± 7.41^b^	308	57.8 vs. 46.8	TAVI	9
Miles, 2016 [39]	UK	No	Cohort	77.8 ± 7.8 vs. 81.5 ± 6.4^b^	88	75 vs. 66	TAVI	7
McDonald, 2015 [40]	USA	No	Matched cohort	71 (58, 80) vs. 70 (58, 79)^c^	1014	48.7 vs. 44.2	Endovascular thrombectomy	9
Mayr, 2016 [41]	Germany	No	RCT	80 (75, 84) vs. 84 (79, 86)^c^	62	41.9 vs. 58.1	TAVI	^𝛿^
Löwhagen, 2017 [42]	Sweden	No	RCT	73 (65, 80) vs. 72 (66, 82)^c^	90	58 vs. 51	Endovascular thrombectomy	^𝛿^
Kleinecke, 2021 [43]	Germany	Yes	Matched cohort	77.0 ± 6.8 vs. 78.0 ± 7.3^b^	311	63.5 vs. 51	Transcatheter left atrial appendage closure	7
Kislitsina, 2019 [44]	USA	No	Cohort	81.9 ± 10.6 vs. 81.2 ± 9.2^b^	286	NA	TAVR	7
Kiramijyan, 2016 [45]	USA	No	Cohort	81.3 ± 10.6 vs. 82.9 ± 7.6^b^	533	50 vs. 50.6	TAVR	8
Jumaa, 2010 [46]	USA	No	Cohort	66.47 vs. 66.6^e^	126	41.5 vs. 53.4	Endovascular Acute Stroke Therapy	8
John, 2014 [47]	USA	No	Cohort	64.8 ± 17 vs. 69.1 ± 13.5^b^	190	40.7 vs. 46.5	Intra-arterial thrombolysis	8
Jadhav, 2017 [48]	USA	No	Matched cohort	67 (55.5, 78.5) vs. 69 (60, 75)^c^	122	41 vs. 54.1	Endovascular Acute Stroke Therapy	9
Hyman, 2017 [49]	USA	Yes	Cohort	81.8 ± 8.4 vs. 82.4 ± 8.2^b^	10,997	53.7 vs. 54.2	TAVR	8
Husser, 2018 [50]	Germany	Yes	Matched cohort	81 ± 5 vs. 81 ± 6^b^	5248	41.8 vs. 41.4	TAVR	9
Herrmann, 2021 [51]	USA	Yes	Cohort	80.5 ± 7.3 vs. 76.6 ± 7.4^b^	1443	53.8 vs. 61.6	TAVR	7
Haurand, 2022 [52]	Germany	Yes	Cohort	80 (76, 84) vs. 81 (77, 83)^c^	104	50.0 vs. 35	TAVR	7
Harjai, 2020 [53]	Australia	Yes	Cohort	82 (77, 87) vs. 83 (77, 87)^c^	477	51.1 vs. 49.2	TAVI	7
Griessenauer, 2017 [54]	USA	Yes	Matched cohort	60.5 (29, 76) vs. 60 (29, 80)^c^	140	14.3 vs. 14.3	Flow diversion for cerebral aneurysm	7
Feil, 2021 [55]	Germany	No	Cohort	73.1 ± 13.1 vs. 73.4 ± 13.0^b^	6103	49.1 vs. 49.4	Endovascular thrombectomy	7
Du, 2020 [56]	China	No	Cohort	60 ± 10 vs. 60 ± 11^b^	178	87.1 vs. 74.4	Endovascular thrombectomy	8
D'Errigo, 2016 [57]	Italy	Yes	Matched cohort	82.0 ± 5.4 vs. 82.7 ± 5.8^b^	620	61.9 vs. 64.5	TAVR	7
Cappellari, 2020 [58]	Italy	Yes	Cohort	72 (59, 79) vs. 74 (63, 81)^c^	3298	55 vs. 47.2	Endovascular thrombectomy	8
Ben-Dor, 2012 [59]	USA	No	Cohort	83.7 ± 7.9 vs. 84.1 ± 5.1^b^	92	36.4 vs. 41.4	TAVR	8
Althoff, 2021 [60]	USA	No	Cohort	65 (52, 77) vs. 65 (53, 78)^c^	17,538	50.5 vs. 46.5	ERCP	9
Yamamoto, 2013 [61]	France	No	Cohort	84.7 ± 7.0 vs. 83.7 ± 7.1^b^	174	46.7 vs. 39.5	TAVI	8
Hoefnagel, 2023 [62]	USA	No	Cohort	65 (55, 75) vs. 68 (60, 76)^c^	125	50 vs. 59.5	Endovascular thrombectomy	8
Reda, 2012 [63]	Australia	No	Cohort	83.4 ± 0.6 vs. 82.6 ± 1.2^b^	74	54.5 vs. 34.1	TAVI	8
Neumann, 2020 [64]	UK	Yes	Cohort	81.3 ± 6.60 vs. 81.9 ± 6.72^b^	1694	51.6 vs. 50.2	TAVR	7
Skutecki, 2022 [65]	France	Yes	Matched cohort	68 ± 13 vs. 68 ± 13^b^	258	63.6 vs. 61.2	Endovascular thrombectomy	9
Liang, 2021 [66]	China	No	Cohort	75.74 ± 7.05 vs. 76.95 ± 5.61^b^	134	66.7 vs. 58.9	TAVR	8
Valente, 2021 [67]	Portugal	No	Cohort	79 (71, 84) vs. 82 (76, 85)^c^	107	54.2 vs. 30.1	TAVI	7
Kanda, 2022 [68]	Japan	No	Cohort	61.9 ± 13.6 vs. 81.6 ± 10.9^b^	101	49 vs. 29	Minimally invasive mitral valve surgery	7
Holmes, 2022 [69]	USA	No	Cohort	77 vs. 77.6^e^	166	97.6 vs. 97.6	TAVR	8
Liang, 2022 [70]	China	No	RCT	64 ± 11 vs. 60 ± 13^b^	87	76.7 vs. 86.4	Endovascular thrombectomy	^𝛿^
Maurice, 2022 [71]	France	Yes	RCT	70.8 ± 13.0 vs. 72.6 ± 12.3^b^	345	52.7 vs. 56.3	Endovascular thrombectomy	^𝛿^
Sanders, 2021 [72]	USA	No	Cohort	81 (74, 86) vs. 80 (73, 86)^c^	79	36.8 vs. 53.7	TAVR	8
Monaco, 2022 [73]	Italy	No	Matched cohort	73 (68, 78) vs. 72 (67–76)^c^	84	71 vs. 69	Endovascular repair of thoracic-abdominal aortic aneurysms	9
Goren, 2015 [74]	Israel	No	Cohort	83 ± 5.5 vs. 83 ± 5.4^b^	204	36 vs. 40	TAVI	8
Aslan, 2021 [75]	Turkey	No	Cohort	77.8 ± 6.9 vs. 78.1 ± 8.9^b^	72	31 vs. 30	TAVI	8

*TAVI *Transcatheter Aortic Valve Implantation, *TAVR *Transcatheter Aortic Valve Replacement, *ERCP *Endoscopic Retrograde Cholangial-Pancreatography

^a^Presented as general anesthesia group vs. sedation group

^b^Means±standard deviation

^c^Median (interquartile range)

^d^Means (95%CI)

^e^Means

𝛿Assessment by using the Cochrane risk of bias criteria. NA: Not reported

### GA versus sedation in all-cause mortality

In our analysis, we examined the impact of sedation and GA on all-cause mortality across five distinct time points according to the data included in this study: 24-h mortality, in-hospital mortality, 30-day mortality, 90-day mortality, and 1-year mortality.

### Mortality at 24 h postoperatively

Only one study [20] reported mortality 24 h postoperatively, suggesting no significant difference between sedation and GA for cardiac catheterization in children under 2 years old.

#### In-hospital mortality

Among the studies included in our analysis, a total of 26 articles were examined to assess in-hospital mortality. These articles consisted of 2 RCTs and 13 cohort studies focusing on cardiac and macrovascular surgery, 4 RCTs, and 7 cohort studies pertaining to cerebrovascular surgery.

**Fig. 2:**
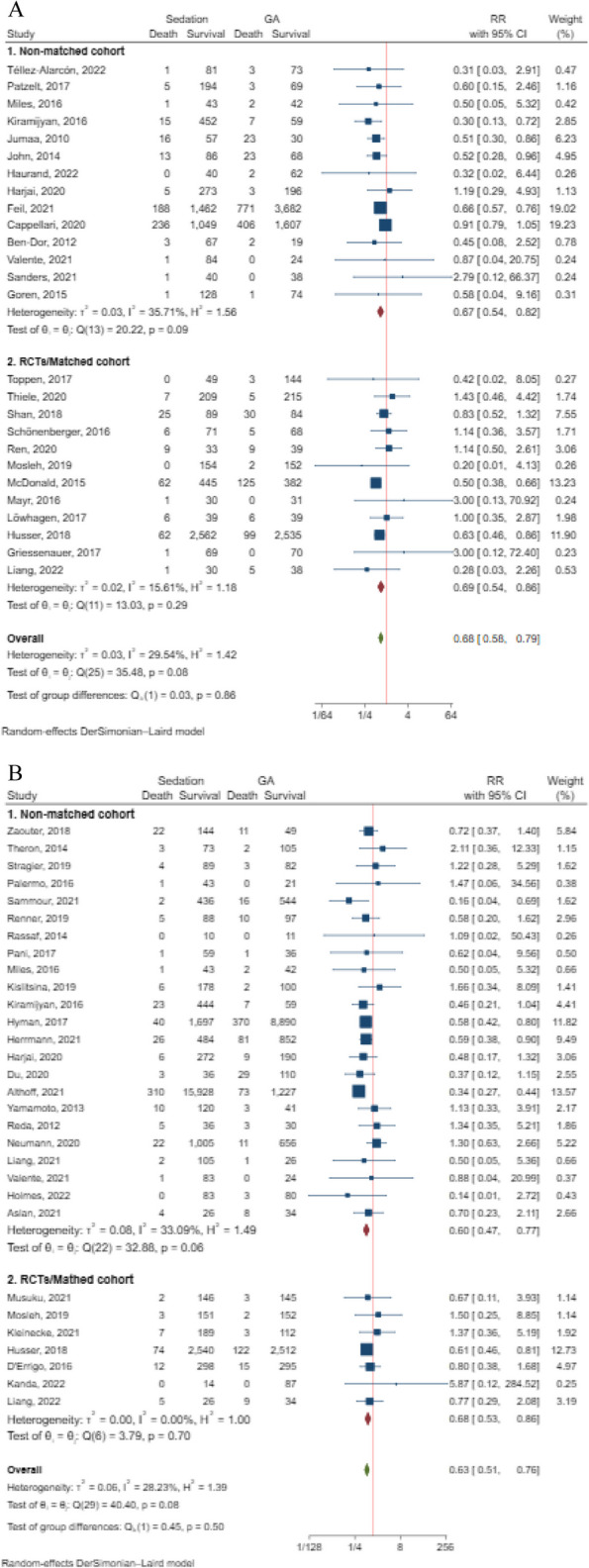
**A** Sedation versus GA on in-hospital mortality (RR = 0.68, 95% CI: 0.58 to 0.79, 19,245 patients) with the subgroup analysis of study design; **B** Sedation versus GA on 30-day mortality (RR = 0.63, 95% CI: 0.51 to 0.76, 42,888 patients) with the subgroup analysis of study design

The pooled results from these studies indicated a significantly lower risk of death associated with the use of sedation compared to GA (RR = 0.68, 95% CI: 0.58 to 0.79, *p* < 0.001) among a total of 19,245 patients (Fig. 2A). Sensitivity analyses further supported these findings (Supplemental Fig. 2), and the level of heterogeneity was not statistically significant (I^2^ = 29.54%, *p* = 0.08).

Subgroup analysis based on study design also demonstrated a lower mortality risk with sedation, regardless of whether it was a non-matched cohort study (RR = 0.67, 95% CI: 0.54 to 0.82) or a matched cohort/RCT (RR = 0.69, 95% CI: 0.54 to 0.86). Notably, the subgroup of matched cohort/RCT showed lower heterogeneity (I^2^ = 15.61%, *p* = 0.29) (Fig. 2A). When examining the effect of surgery type, the results indicated that patients undergoing cardiac and macrovascular or cerebrovascular surgery had a lower risk of death with sedation (cardiac and macrovascular: RR = 0.56, 95% CI: 0.46 to 0.67; cerebrovascular: RR = 0.75, 95% CI: 0.62 to 0.92) (Supplemental Fig. 3). Additionally, Egger’s test showed no significant publication bias (t = 0.55, *p* = 0.588), and the funnel plot is presented in Supplemental Fig. 4.

**Fig. 3:**
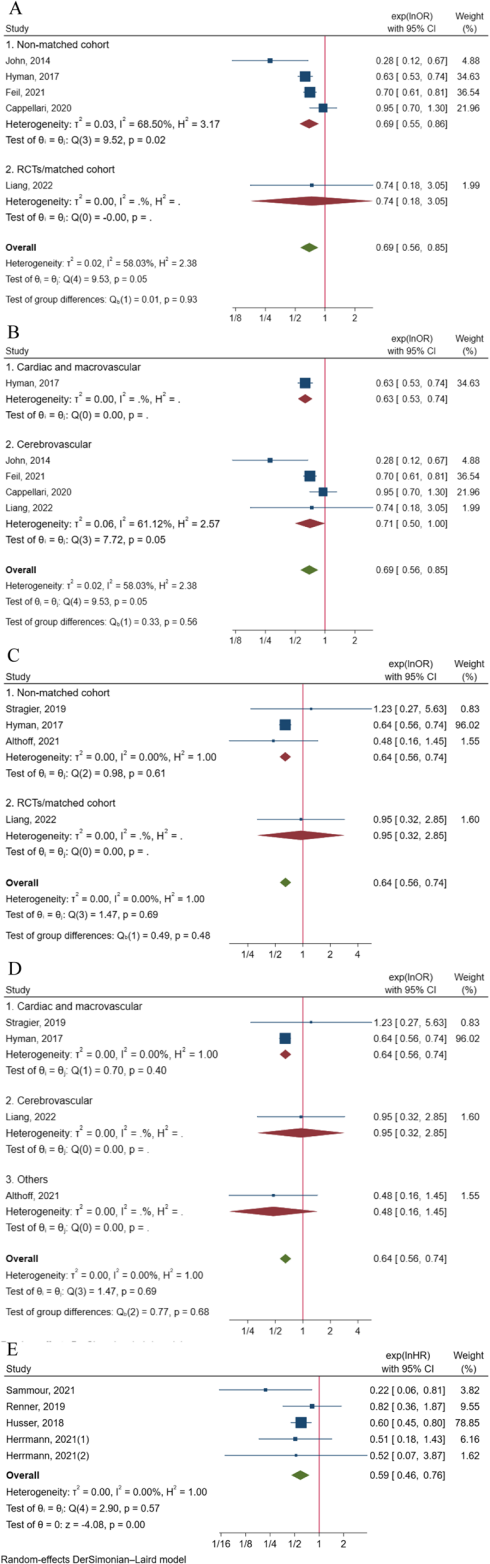
**A** Pooled result of adjusted effect size (OR) of sedation on the risk of in-hospital death with the subgroup analysis of study design; **B** Pooled result of adjusted effect size (OR) of sedation on the risk of in-hospital death with the subgroup analysis of type of surgery; **C** Pooled result of adjusted effect size (OR) of sedation on the risk of 30-day death with the subgroup analysis of study design; **D** Pooled result of adjusted effect size (OR) of sedation on the risk of 30-day death with the subgroup analysis of type of surgery; **E** Pooled result of adjusted effect size (HR) of sedation on the risk of 30-day death

Moreover, five articles provided adjusted effect sizes in the form of odds ratios (ORs) for in-hospital mortality [47, 49, 55, 58, 70]. The pooled results of the five adjusted OR values demonstrated that sedation was associated with a lower risk of in-hospital death compared to GA (RR = 0.69, 95% CI: 0.56 to 0.85, *p* < 0.001) among a total of 20,675 patients, although there was significant heterogeneity (I^2^ = 58.03%, *p* = 0.05) (Fig. 3A). Notably, subgroup analysis limited to non-matched cohort studies showed consistent results (RR = 0.69, 95% CI: 0.55 to 0.86, I^2^ = 68.50%) (Fig. 3A). However, the subgroup analysis specific to cerebrovascular surgery revealed an approximately non-significant result (RR = 0.71, 95% CI: 0.50 to 1.00, *p* = 0.05, I^2^ = 61.12%) (Fig. 3B).

#### 30-day mortality

A total of 30 articles were included in our analysis to investigate 30-day mortality in the comparison between sedation and GA. These articles consisted of 27 cohort studies focusing on cardiac and macrovascular surgery, 1 RCT and 1 cohort study pertaining to cerebrovascular surgery, and 1 non-matched cohort study on ERCP surgery.

The pooled analysis revealed a significantly lower risk of death within 30 days postoperatively in the sedation group compared to the GA group (RR = 0.63, 95% CI: 0.47 to 0.77, *p* < 0.001), involving a total of 42,888 patients. There was no significant heterogeneity observed among the included studies (I^2^ = 28.23%, *p* = 0.08) (Fig. 2B). Sensitivity analyses, conducted by sequentially excluding individual studies, consistently supported these findings, indicating the robustness of the evidence (Supplemental Fig. 5).

Subgroup analysis focused on matched cohort or RCTs (RR = 0.68, 95% CI: 0.53 to 0.86, I^2^ = 0%) (Fig. 2B) and subgroup analysis specific to cardiac and macrovascular surgery (RR = 0.66, 95% CI: 0.56 to 0.77, I^2^ = 0%) (Supplemental Fig. 6) revealed no significant heterogeneity and provided insights into the possible sources of significant heterogeneity observed in the overall analysis. Egger’s test (t = 2.68, *p* = 0.012) and the funnel plot (Supplemental Fig. 7) suggested the presence of publication bias concerning the outcome of 30-day mortality. However, the subgroup of cerebrovascular surgery, involving only two studies, revealed no difference between the two groups (RR = 0.56, 95% CI: 0.27 to 1.18, I^2^ = 0%).

Regarding the adjusted effect size for the risk of 30-day mortality, 8 articles reported adjusted effect sizes, with four articles [26, 49, 60, 70] presenting OR values and four articles [7, 30, 50, 51] presenting HR. The pooled analysis of the OR values indicated a significant difference between the sedation and GA groups (RR = 0.64, 95% CI: 0.56 to 0.74, *p* < 0.001), involving a total of 28,787 patients, with non-significant heterogeneity (I^2^ = 0%, *p* = 0.69) (Fig. 3C). The subgroup analysis limited to cohort studies consistently showed significant differences (RR = 0.64, 95% CI: 0.56 to 0.74) (Fig. 3C). In the subgroup analysis of the types of surgery, the number of literatures for each subgroup was small, and more than one literature was integrated only in the subgroup of cardiac and macrovascular surgery, showing that patients with sedation had a lower risk of death (RR = 0.64, 95% CI: 0.56 to 0.74) (Fig. 3D). Meanwhile, the pooled analysis of the HR values demonstrated a lower risk of death in the sedation group compared to the GA group (RR = 0.59, 95% CI: 0.46 to 0.76, *p* < 0.001), involving 7,889 patients, with non-significant heterogeneity (I^2^ = 0%, *p* = 0.57) (Fig. 3E).

#### 90-day mortality

In our analysis examining the influence of sedation compared to GA on the risk of 90-day mortality, a total of 12 studies were incorporated. It is noteworthy that the majority of these studies focused on cerebrovascular surgery, encompassing 6 randomized RCTs and 4 cohort studies. However, there was limited representation for other surgical procedures, with only one cohort study investigating the comparison of cardiac and macrovascular surgery, and another cohort study reporting on the comparison of ERCP surgery.

**Fig. 4:**
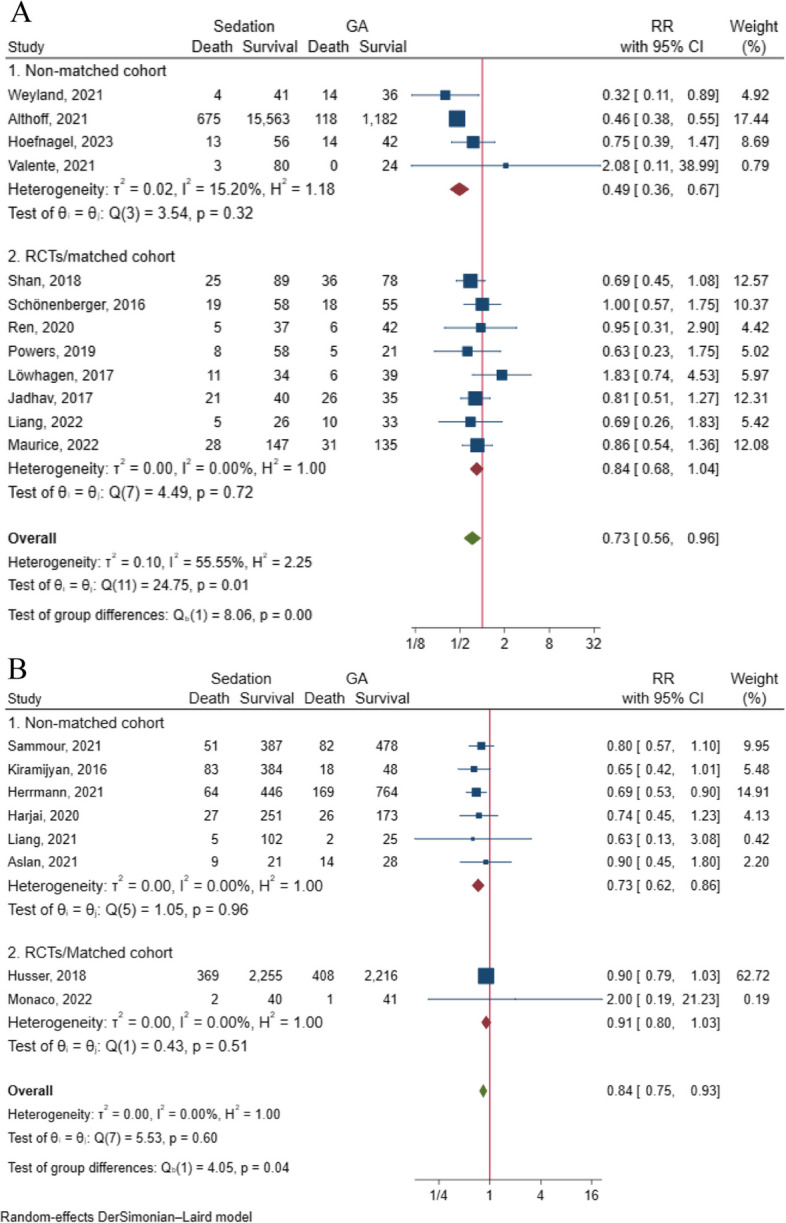
**A** Sedation versus GA on 90-day mortality (RR = 0.73, 95% CI: 0.56 to 0.96, 19,052patients) with the subgroup analysis of study design; **B** Sedation versus GA on one-year mortality (RR = 0.91, 95% CI: 0.83 to 1.03, 8,989 patients) with the subgroup analysis of study design

The pooled results revealed a significant difference in the risk of postoperative 90-day death between the two groups (RR = 0.73, 95% CI: 0.56 to 0.96, *p* = 0.02), involving a total of 19,052 patients, with significant heterogeneity (I^2^ = 55.55%, *p* = 0.01) (Fig. 4A). Subgroup analysis based on study design demonstrated disparate outcomes between cohort and matched cohort/RCTs groups (cohort: RR = 0.49, 95% CI: 0.36 to 0.67; matched cohort/RCT: RR = 0.84, 95% CI: 0.68 to 1.04), indicating non-significant heterogeneity within both groups (Fig. 4A). Furthermore, subgroup analysis specific to cerebrovascular surgery highlighted a difference in the risk of postoperative 90-day death between the two groups (RR = 0.80, 95% CI: 0.66 to 0.98, I^2^ = 0%) (Supplemental Fig. 8). However, sensitivity analysis revealed that the removal of two studies [22, 27] could impact the conclusion (Supplemental Fig. 9). Additionally, the funnel plot (Supplemental Fig. 10) and Egger’s test (t = 2.65, *p* = 0.024) indicated the presence of publication bias, highlighting the limitations of the evidence.

**Fig. 5:**
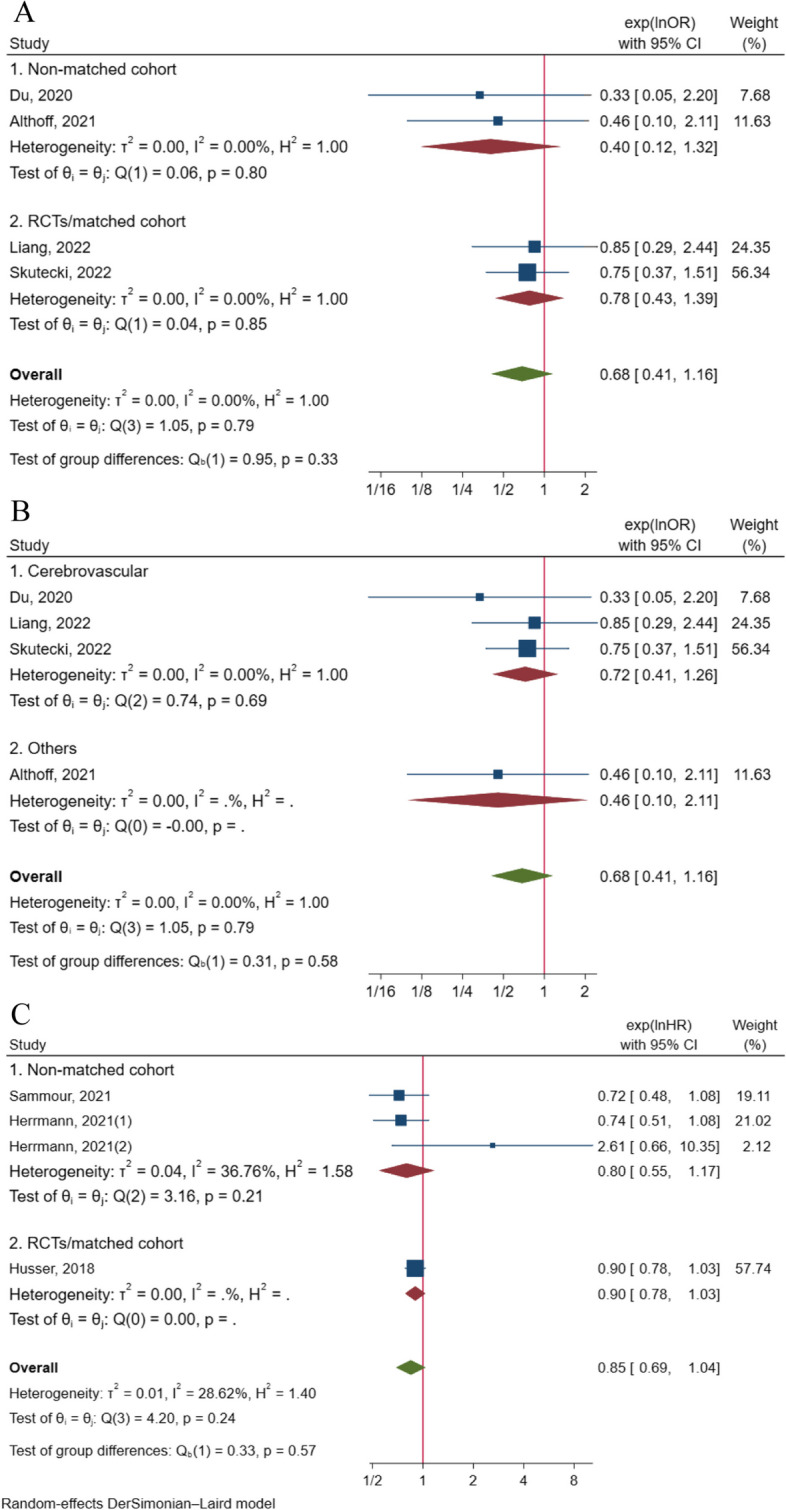
**A** Pooled result of adjusted effect size (OR) of sedation on the risk of 90-day death with the subgroup analysis of study design; **B** Pooled result of adjusted effect size (OR) of sedation on the risk of 90-day death with the subgroup analysis of type of surgery; **C** Pooled result of adjusted effect size (HR) of sedation on the risk of one-year death with the subgroup analysis of study design

Four articles [56, 60, 65, 70] reported the adjusted effect size regarding the relationship between sedation/GA and 90-day mortality, all of which provided OR values. In contrast to the pooled results of deaths, the pooled analysis of the effect values showed no significant difference between the two groups (RR = 0.68, 95% CI: 0.41 to 1.16, *p* = 0.16), involving a total of 18,061 patients, with non-significant heterogeneity (I^2^ = 0%, *p* = 0.79). Subgroup analyses based on study design and surgery type were consistent with these results (Fig. 5A, B).

#### One-year mortality

In our analysis investigating the relationship between sedation and one-year mortality, a total of 8 articles were included. It is important to note that all of these articles focused exclusively on cohort studies pertaining to cardiac and macrovascular surgery. While the available evidence provides insights into the impact of sedation on one-year mortality in this specific surgical context, there is currently limited data available for other types of surgeries.

The pooled results revealed a correlation between sedation and a lower risk of one-year death compared to GA (RR = 0.84, 95% CI: 0.75 to 0.93, *p* < 0.001), involving a total of 8,989 patients, with non-significant heterogeneity (I^2^ = 0%, *p* = 0.60) (Fig. 4B). While the cohort study design consistently showed a lower risk of one-year mortality with sedation compared to GA (RR = 0.73, 95% CI: 0.62 to 0.86, I^2^ = 0%), the effect became non-significant for matched cohort/RCT in the subgroup analysis (RR = 0.91, 95% CI: 0.80 to 1.03, I^2^ = 0%) (Fig. 4B). However, data pooling was not possible for the analysis of surgery-type subgroups.

The adjusted effect size between sedation and the risk of one-year death was examined in 3 studies. The pooled result of these 3 articles, all of which provided HR values, demonstrated no significant difference in the risk of one-year death between the two groups (RR = 0.85, 95% CI: 0.69 to 1.04, *p* = 0.11), involving a total of 7,689 patients, with non-significant heterogeneity (I^2^ = 28.62%, *p* = 0.24). Subgroup analysis of cohort studies also found consistent results (RR = 0.80, 95% CI: 0.55 to 1.17, I^2^ = 36.76%) (Fig. 5C). However, no data pooling was possible for the effect size analysis of surgery-type subgroups.

#### Certainty of evidence

The level of certainty regarding the evidence for each outcome was presented in Supplementary Table 3. The overall certainty of the evidence was classified as low for three outcomes, namely the risk of in-hospital mortality based on pooled number of cases, the risk of one-year mortality based on pooled number of cases, and the effect values. For the remaining outcomes, the overall certainty was considered very low. The primary factors contributing to the downgrade in evidence included: (a) the inclusion of observational data; (b) a high I^2^ value exceeding 30%; and (c) the presence of significant publication bias.

## Discussion

**Fig. 6:**
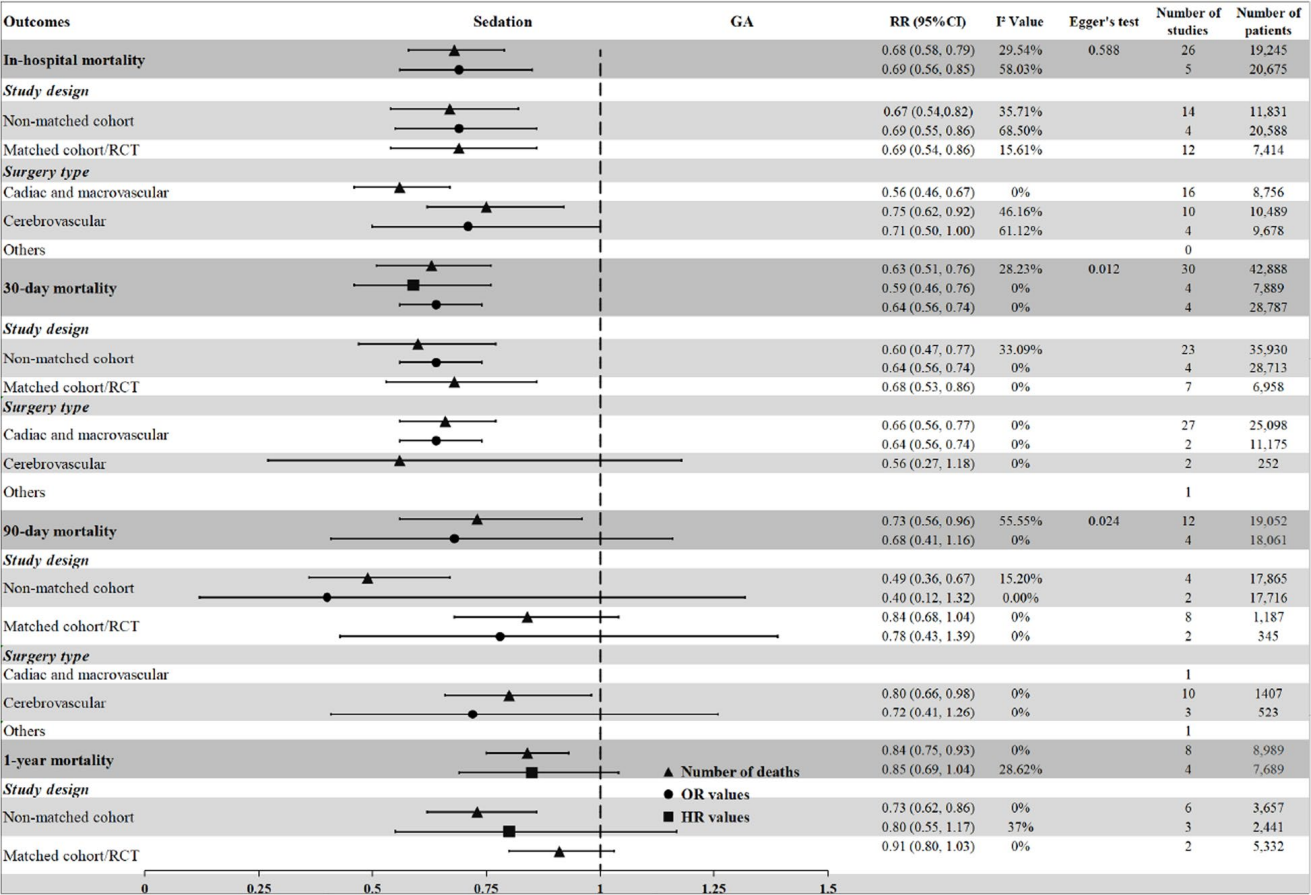
Summary of pooled results on all-cause mortality across four distinct time points (in-hospital, 30-day, 90-day, and one-year) and subgroup analyses

This systematic review and meta-analysis, including both RCTs and cohort studies and pooling outcomes of mortality data and effect size (ORs and HRs), compared the all-cause mortality after sedation versus GA. In-hospital mortality: Sedation was associated with a reduced risk of in-hospital death, regardless of whether patients underwent percutaneous cardiac and macrovascular surgery or cerebrovascular surgery. 30-day mortality: Sedation showed lower risks of death within 30 days postoperatively in patients undergoing percutaneous cardiac and macrovascular surgery, but not in those undergoing cerebrovascular surgery. 90-day and one-year mortality: Discrepancies were observed between the results from pooled mortality and those from effect size analysis (ORs and HRs) for both 90-day and one-year mortality. Subgroup analyses based on different study types and surgery types also yielded varying results (Fig. 6).

Our search strategy led us to focus on three specific procedures: cardiac and macrovascular surgery, cerebrovascular surgery, and ERCP. These were chosen due to the prevalence of sedation or general anesthesia (GA) usage in endovascular and gastrointestinal interventional procedures. The decision to administer sedation or GA is influenced by various factors, including procedural accuracy, anesthesiologist-rated risk, surgeon preference, and procedure-specific considerations. Our study's results suggest a potential slight superiority of sedation over GA in terms of in-hospital, and 30-day all-cause mortality. However, the advantage of sedation over GA at long-term postoperative time points remains unclear or ambiguous.

Prior meta-analyses have predominantly focused on specific surgery types, such as cardiovascular [76–79] or cerebrovascular surgery [10, 80–83], or specific research designs, such as RCTs [82–84]. While these approaches allowed for a high-quality and targeted comparison between sedation and GA, they often suffer from limitations due to a smaller number of included articles and limited data, resulting in an incomplete comparison between them.

Two previous meta-analyses [77, 85] specifically examined the risk of in-hospital death in transcatheter heart valve surgery (TAVI or TAVR) and consistently reported no significant difference in mortality rates between sedation and GA. However, our pooled results, encompassing a broader range of studies and incorporating articles published after the cutoff date of those meta-analyses, demonstrated a lower risk of death associated with sedation compared to GA. We speculate that advancements in surgical techniques and anesthesia technology, along with the inclusion of high-quality studies, may contribute to the discrepancies between our findings and previous analyses. Additionally, the broad inclusion and exclusion criteria employed in these studies aimed to increase the number of eligible articles, but this may have led to comparisons that did not strictly adhere to sedation versus GA standards. For instance, the meta-analysis of Ehret C et al. [85] included clinical studies [86, 87] that compared surgical methods rather than anesthesia type, despite both procedures being performed under sedation and GA. Furthermore, our sensitivity analysis and consistent results from overall and subgroup analyses provide robustness to our findings.

About 30-day mortality, previous meta-analyses primarily focused on transcatheter heart valve surgery (TAVI or TAVR) and yielded conflicting findings. Some studies reported no significant difference between minimal anesthesia care and GA, while others suggested a lower overall 30-day mortality with sedation. For instance, a meta-analysis encompassing seven observational studies and a total of 1,542 patients reported no significant difference in overall 30-day mortality between MAC and GA (RR = 0.77, 95% CI 0.38 to 1.56; *P* = 0.46) based on a literature search spanning from January 1, 2005, to January 31, 2013 [78]. Similarly, other meta-analyses conducted around the time of publication of the aforementioned study produced consistent results [85, 88].

However, two recent meta-analyses demonstrated that the use of sedation for TAVR was associated with a lower overall 30-day mortality (RR = 0.73, 95% CI: 0.57 to 0.93; *P* = 0.01) through the pooling of mortality data [79, 89]. In alignment with these findings, our pooled analysis of the number of deaths also revealed a reduced risk of death in the sedation group compared to the GA group. The discrepancy between these findings could be attributed to advancements in surgical techniques and anesthesia technology over time. Furthermore, in a more accurate approach than previous studies, we integrated risk estimates based on ORs and HRs, indicating consistent evidence regarding the lower risk of 30-day mortality of sedation. However, it is important to note that our subgroup analysis specific to cerebrovascular surgery did not reveal a difference in in-hospital mortality between sedation and GA. Given the limited number of studies available for this analysis, caution should be exercised when interpreting these results.

Regarding 90-day mortality, previous meta-analyses focused on transcatheter cerebrovascular surgery consistently showed no reduced risk of mortality with sedation compared to GA [83, 84, 90]. Our subgroup analysis of matched cohorts or RCTs also supported this conclusion, as did the pooling of OR values. However, our overall analysis of pooled the number of deaths did reveal a difference in 90-day mortality. Nonetheless, the effect size was minimal, and significant heterogeneity and serious publication bias limited the interpretation of this difference. Therefore, caution should be exercised when interpreting these findings.

To our knowledge, this is the first meta-analysis conducting the comparison in one-year mortality, involving only 8 articles focusing on cardiac and macrovascular surgery. However, we observed inconsistencies between the results obtained from different pooling methods: number of deaths and HR. Given that the included papers consisted mainly of retrospective observational studies, with only two matched cohort studies, the presence of numerous confounding factors is inevitable. Consequently, the adjusted effect values (HR) provided a more reliable indication of no difference between sedation and GA, aligning with the majority of clinical studies [45, 53, 66, 75].

Although the mechanism by which sedation reduces the risk of postoperative death remains uncertain, previous studies offered some insights. Firstly, sedation leads to a reduction in the dosage of anesthetic drugs, thereby mitigating cardiac depression and periprocedural hemodynamic instability [42]. This reduction may decrease the risk of permanent neurological deficits, myocardial ischemia, and renal impairment during the surgical period [91, 92]. Secondly, sedation obviates the need for tracheal intubation, which is associated with an increased risk of intraoperative complications, particularly pulmonary complications [55, 93, 94]. Furthermore, avoiding tracheal intubation eliminates the need to transfer intubated patients to the ICU [74, 95], thereby reducing the patient's susceptibility to postoperative infections.

However, these clues are more closely related to early postoperative mortality, rather than mid- or long-term postoperative mortality. Just like the indication of our results, the effect of the type of anesthesia on these terms was more ambiguous, especially 90-day and one-year mortality. As everyone knows, the risk of postoperative long-term death is related to more factors compared to early one [22, 96], such as home nursing, work, and living habits after discharge, compliance with medical instructions, etc., and anesthesia choice is only a point-in-time intervention, it is hard to imagine how the choice of a single point can affect long-term outcomes. At the same time, no previous studies have established a real and reliable association between anesthesia choice and the risk factors for long-term postoperative death. In our analysis, a lower risk of one-year death was associated with sedation compared to GA, however, all studies included in this analysis were cohort studies, and the inconsistency between the results of the matched cohort subgroup analysis and the main analysis suggested no difference between sedation and GA. For randomized controlled studies, follow-up of up to one year is difficult, which also contributes to the lack of such studies. We seem to get some clues from similar previous RCTs that in elderly patients having hip fracture surgery with spinal anesthesia supplemented with propofol sedation, heavier intraoperative sedation was not associated with significant differences in mortality or return to pre-fracture ambulation up to one year after surgery [97].

### Strengths and limitations

This meta-analysis provides a comprehensive synthesis of the available evidence on the association between sedation and GA with all-cause mortality. We have examined a broad spectrum of postoperative mortality outcomes, spanning various time points (24 h postoperatively, in-hospital, 30-day, 90-day, and one-year), and have employed diverse statistical analysis methods including ORs and HRs. Through separate pooling of these outcomes, our aim is to move beyond single time point analyses and individual outcomes, allowing for a nuanced exploration of the relationship between anesthesia type and all-cause mortality, thus facilitating a comprehensive comparison between sedation and GA.

However, it is crucial to acknowledge the limitations of our meta-analysis. Firstly, to comprehensively evaluate all-cause mortality between sedation and GA, we included studies with diverse designs, including retrospective observational studies, which inherently contribute to a lower level of evidence for our findings, all of which were graded as very low to low certainty. Furthermore, despite employing various analytical approaches, we encountered significant heterogeneity and conflicting results, particularly in the assessment of 90-day and one-year postoperative mortality. Thirdly, our study focused on patients undergoing percutaneous procedures, with a predominant emphasis on endovascular surgery. Therefore, the generalizability of our conclusions to all surgical patients eligible for sedation or GA is limited. Lastly, the studies included in our analysis employed different anesthesia techniques (e.g., total intravenous or intravenous-inhalational anesthesia, intubation or laryngeal mask general anesthesia) or sedation techniques (e.g., conscious sedation, deep sedation, and MAC), which could further increase heterogeneity and robustness of pooled outcomes. Consequently, future research should aim to explore the comparison between sedation and GA of different types across a broader range of postoperative outcomes, particularly in non-endovascular surgery and long-term outcomes, through high-quality clinical studies.

## Conclusion

The currently available evidence, graded as very low to low certainty, suggests a possible slight advantage of sedation over GA in reducing the risk of in-hospital mortality, specifically in cardiac and macrovascular surgery. However, the extent of this difference is not clearly evident or uncertain in the medium and long-term postoperative periods. Furthermore, the comparison between sedation and GA in cerebrovascular surgery and other surgical patient populations also yields uncertain results, despite the limited number of studies included in the subgroup analysis.

### Supplementary Information

**Supplementary Material 1.**

**Supplementary Material 2.**

**Supplementary Material 3.**

**Supplementary Material 4.**

## Data Availability

All data relevant to the study are included in the article or uploaded as supplementary information.

## Abbreviations

GA: General anesthesia
TAVR: Transcatheter aortic valve replacement
MAC: Monitored anesthesia care
RCTs: Randomized controlled trials
ORs: Odds ratios
HRs: Hazard ratios
RRs: Risk ratios
CIs: Confidence interval
PRISMA: Preferred reporting items for systematic reviews and meta-analyses statement
