# Supplementary material for: Sedation versus general anesthesia on all-cause mortality in patients undergoing percutaneous procedures: a systematic review and meta-analysis
Source: BMC Anesthesiol. 2024 Apr 2;24:126. doi: 10.1186/s12871-024-02505-w (PMC10985877; doi:10.1186/s12871-024-02505-w)
Supplement: Supplementary file 1 — Supplementary Material 1. [file 12871_2024_2505_MOESM1_ESM.pdf]

Supplementary Table 1 Search strategy

Online database	Search number	Search strategy	Number of articles
PubMed	#1	general anesthesia[MeSH Terms]	61639
	#2	("general anesthesia"[Title/Abstract]) OR ("inhalation anesthesia"[Title/Abstract]) OR ("inhalational anesthesia"[Title/Abstract]) OR ("insufflation anesthesia"[Title/Abstract]) OR ("rebreathing anesthesia"[Title/Abstract]) OR ("balanced anesthesia"[Title/Abstract]) OR ("closed circuit anesthesia"[Title/Abstract]) OR ("intratracheal anesthesia"[Title/Abstract]) OR ("endotracheal anesthesia"[Title/Abstract]) OR ("rectal anesthesia"[Title/Abstract])	47231
	#3	("general anaesthesia"[Title/Abstract]) OR ("inhalation anaesthesia"[Title/Abstract]) OR ("inhalational anaesthesia"[Title/Abstract]) OR ("insufflation anaesthesia"[Title/Abstract]) OR ("rebreathing anaesthesia"[Title/Abstract]) OR ("balanced anaesthesia"[Title/Abstract]) OR ("closed circuit anaesthesia"[Title/Abstract]) OR ("intratracheal anaesthesia"[Title/Abstract]) OR ("endotracheal anaesthesia"[Title/Abstract]) OR ("rectal anaesthesia"[Title/Abstract])	17114
	#4	#1 OR #2 OR #3	103021
	#5	(deep sedation[MeSH Terms]) OR (conscious sedation[MeSH Terms])	10769
	#6	(sedation*[Title/Abstract]) OR ("monitored anesthesia care"[Title/Abstract]) OR ("monitored anaesthesia care"[Title/Abstract]) OR (MAC[Title/Abstract])	65054
	#7	#5 OR #6	67773
	#8	"mortality"[MeSH Terms] OR "death"[MeSH	2953997

		Terms] OR "survival"[MeSH Terms] OR "mortality"[Title/Abstract] OR "death"[Title/Abstract] OR "fatal"[Title/Abstract] OR "survival"[Title/Abstract] OR "survive"[Title/Abstract] OR "deaths"[Title/Abstract] OR "mortalities"[Title/Abstract] OR "fatality"[Title/Abstract]	
	#9	#4 AND #7 AND #8	727
EMBASE	#1	'general anesthesia'/exp	112,816
	#2	'general anesthesia':ab,ti OR 'general anaesthesia':ab,ti OR 'inhalation anesthesia':ab,ti OR 'inhalational anesthesia':ab,ti OR 'inhalation anaesthesia':ab,ti OR 'inhalational anaesthesia':ab,ti OR 'balanced anesthesia':ab,ti OR 'balanced anaesthesia':ab,ti OR 'colsed circuit anaesthesia':ab,ti OR 'closed circuit anesthesia':ab,ti OR 'insufflation anesthesia':ab,ti OR 'insufflation anaesthesia':ab,ti OR 'intratracheal anesthesia':ab,ti OR 'intratracheal anaesthesia':ab,ti OR 'endotracheal anesthesia':ab,ti OR 'endotracheal anaesthesia':ab,ti OR 'rectal anaesthesia':ab,ti OR 'rectal anesthesia':ab,ti OR 'rebreathing anesthesia':ab,ti OR 'rebreathing anaesthesia':ab,ti	91,488
	#3	#1 OR #2	145,153
	#4	'conscious sedation'/exp	9629
	#5	'deep sedation'/exp	3722
	#6	sedation*:ab,ti OR 'monitored anesthesia care':ab,ti OR 'monitored anaesthesia care':ab,ti OR MAC:ab,ti	101,505
	#7	#4 OR #5 OR #6	104,950
	#8	'all cause mortality'/exp	63,083

	#9	'death'/exp	819,573
	#10	'survival'/exp	1,374,146
	#11	mortality:ab,ti OR death:ab,ti OR deaths:ab,ti OR fatal:ab,ti OR survival:ab,ti OR survive:ab,ti OR mortalities:ab,ti OR fatality:ab,ti	3,919,054
	#12	#8 OR #9 OR #10 OR #11	4,467,383
	#13	#3 NAD #7 AND#12	1,503
Cochrane Libray	#1	MeSH descriptor: [Anesthesia, General] explode all trees	7414
	#2	(“general anesthesia”):ti,ab,kw OR (“inhalation anesthesia”):ti,ab,kw OR (“inhalational anesthesia”):ti,ab,kw OR (“insufflation anesthesia”):ti,ab,kw OR (“closed circuit anesthesia”):ti,ab,kw OR (“rebreathing anesthesia”):ti,ab,kw OR (“inratracheal anesthesia”):ti,ab,kw OR (“endotracheal anesthesia”):ti,ab,kw OR (“rectal anesthesia”):ti,ab,kw OR (“balanced anesthesia”):ti,ab,kw OR (“general anaesthesia”):ti,ab,kw OR (“inhalation anaesthesia”):ti,ab,kw OR (“inhalational anaesthesia”):ti,ab,kw OR (“insufflation anaesthesia”):ti,ab,kw OR (“closed circuit anaesthesia”):ti,ab,kw OR (“rebreathing anaesthesia”):ti,ab,kw OR (“inratracheal anaethesia”):ti,ab,kw OR (“endotracheal anaesthesia”):ti,ab,kw OR (“rectal anaesthesia”):ti,ab,kw OR (“balanced anaesthesia”):ti,ab,kw	25,880
	#3	#1 OR #2	29,134
	#4	MeSH descriptor: [Deep Sedation] explode all trees	198
	#5	MeSH descriptor: [Conscious Sedation] explode	1559

		all trees	
	#6	(sedation*):ti,ab,kw OR ("monitored anesthesia care"):ti,ab,kw OR ("monitored anaesthesia care"):ti,ab,kw OR (MAC):ti,ab,kw	25,794
	#7	#4 OR #5 OR #6	25,794
	#8	MeSH descriptor: [Mortality] explode all trees	16612
	#9	MeSH descriptor: [Death] explode all trees	2980
	#10	MeSH descriptor: [Survival] explode all trees	165
	#11	(mortality):ti,ab,kw OR (mortalities):ti,ab,kw (death):ti,ab,kw OR (deaths):ti,ab,kw OR (survival):ti,ab,kw OR (survive):ti,ab,kw OR (fatal):ti,ab,kw OR (fatality):ti,ab,kw	244,532
	#12	#8 OR #9 OR #10 OR #11	245,276
	#13	#3 AND #7 AND #12	191
