# Supplementary material for: Sedation versus general anesthesia on all-cause mortality in patients undergoing percutaneous procedures: a systematic review and meta-analysis
Source: BMC Anesthesiol. 2024 Apr 2;24:126. doi: 10.1186/s12871-024-02505-w (PMC10985877; doi:10.1186/s12871-024-02505-w)
Supplement: Supplementary file 2 — Supplementary Material 2. [file 12871_2024_2505_MOESM2_ESM.pdf]

Supplementary Table 3 The overall certainty of evidence

Outcomes	Pooling Data	No. study Comparison	Downgrade quality of evidence					Upgrade quality of evidence			Overall quality of evidence
			Risk of bias	Inconsistency	Imprecision	Indirectness	Publication Bias	Dose-response	Large Effect	Plausible Confounding	
In-hospital mortality	No. of deaths	27	Not serious	Not serious	Not serious	Not serious	Not serious	No	No	No	⊕⊕◯◯ Low
	Effect values	5	Not serious	Serious	Not serious	Not serious	NA	No	No	No	⊕◯◯◯ Very low
30-day mortality	No. of deaths	31	Not serious	Serious	Not serious	Not serious	Not serious	No	No	No	⊕◯◯◯ Very low
	Effect values	8	Not serious	Not serious	Not serious	Not serious	NA	No	No	No	⊕⊕◯◯ Low
90-day mortality	No. of deaths	12	Not serious	Serious	Not serious	Not serious	Serious	No	No	No	⊕◯◯◯ Very low
	Effect values	4	Not serious	Not serious	Serious	Not serious	NA	No	No	No	⊕◯◯◯ Very low
One-year mortality	No. of deaths	8	Not serious	Not serious	Not serious	Not serious	NA	No	No	No	⊕⊕◯◯ Low
	Effect values	4	Not serious	Not serious	Not serious	Not serious	NA	No	No	No	⊕⊕◯◯ Low
