# Supplementary material for: Sedation versus general anesthesia on all-cause mortality in patients undergoing percutaneous procedures: a systematic review and meta-analysis
Source: BMC Anesthesiol. 2024 Apr 2;24:126. doi: 10.1186/s12871-024-02505-w (PMC10985877; doi:10.1186/s12871-024-02505-w)
Supplement: Supplementary file 3 — Supplementary Material 3. [file 12871_2024_2505_MOESM3_ESM.pdf]

Supplementary Table 2 Additional detailed features of sedation and GA in studies

Study	General anesthetic	Airway management during GA	Type of sedation	Drug of sedation
Mikus, 2021(20)	CIIA	Endotracheal intubation	DS	Propofol and ketamine
Zaouter, 2018(21)	TIVA	Endotracheal intubation	Unspecified	Propofol
Weyland, 2021(22)	Unspecified	Unspecified	CS	Unspecified
Toppen, 2017(23)	Unspecified	Unspecified	CS	Unspecified
Thiele, 2020(8)	Unspecified	Endotracheal intubation	CS	Propofol and dexmedetomidine
Theron, 2014(24)	CIIA	Endotracheal intubation / Laryngeal mask	Unspecified	Unspecified
Téllez-Alarcón, 2022(25)	CIIA	Unspecified	CS	Propofol, dexmedetomidine and remifentanil
Stragier, 2019(26)	TIVA	Endotracheal intubation	MAC	Propofol and midazolam
Shan, 2018(27)	TIVA	Endotracheal intubation	CS	Dexmedetomidine and midazolam
Schönenberger, 2016(28)	CIIA	Endotracheal intubation	CS	Propofol, remifentanil, and midazolam
Palermo, 2016(29)	CIIA	Endotracheal intubation	MAC	Dexmedetomidine and fentanyl

Sammour, 2021(7)	CIIA	Unspecified	MAC	Propofol, fentanyl dexmedetomidine and midazolam
Renner, 2019(30)	CIIA	Endotracheal intubation / Laryngeal mask	CS	Propofol and remifentanil
Ren, 2020(31)	TIVA	Endotracheal intubation	CS	Propofol, dexmedetomidine, midazolam and fentanyl
Rassaf, 2014(32)	TIVA	Endotracheal intubation	DS	Propofol and midazolam
Powers, 2019(33)	Unspecified	Unspecified	CS	Unspecified
Piayda, 2021(34)	Unspecified	Unspecified	CS	Unspecified
Patzelt, 2017(35)	TIVA	Endotracheal intubation	DS	Propofol and midazolam
Pani, 2017(36)	CIIA	Endotracheal intubation	MAC	Midazolam, propofol, fentanyl, dexmedetomidine and ketamine
Musuku, 2021(37)	CIIA	Laryngeal mask	MAC	Propofol, midazolam and ketamine
Mosleh, 2019(38)	Unspecified	Endotracheal intubation	CS	Unspecified
Miles, 2016(39)	CIIA	Endotracheal intubation	CS	remifentanil
McDoUnspecifiedld, 2015(40)	Unspecified	Unspecified	CS	Unspecified
Mayr, 2016(41)	TIVA	Endotracheal intubation	Unspecified	Midazolam, propofol and remifentanil

Löwhagen, 2017(42)	CIIA	Endotracheal intubation	CS	Propofol and remifentanil
Kleinecke, 2021(43)	TIVA	Endotracheal intubation	CS	propofol
KislitsiUnspecified, 2019(44)	Unspecified	Endotracheal intubation	MS	Unspecified
Kiramijyan, 2016(45)	Unspecified	Endotracheal intubation	MAC	Propofol, dexmedetomidine, ketamine and midazolam
Jumaa, 2010(46)	Unspecified	Unspecified	CS	Unspecified
John, 2014(47)	Unspecified	Unspecified	MAC	Propofol, dexmedetomidine, midazolam and fentanyl
Jadhav, 2017(48)	CIIA	Endotracheal intubation	MAC	Unspecified
Hyman, 2017(49)	Unspecified	Unspecified	CS	Unspecified
Husser, 2018(50)	Unspecified	Unspecified	CS	Unspecified
Herrmann, 2021(51)	Unspecified	Unspecified	CS	Unspecified
Haurand, 2022(52)	CIIA	Endotracheal intubation	DS	Propofol and midazolam
Harjai, 2020(53)	Unspecified	Endotracheal intubation	CS	Midazolam, fentanyl and dexmedetomidine

GriesseUnspecifieduer, 2017(54)	CIIA	Endotracheal intubation	CS	Fentanyl and midazolam
Feil, 2021(55)	Unspecified	Unspecified	CS	Unspecified
Du, 2020(56)	TIVA	Endotracheal intubation/ Laryngeal mask	CS	propofol
D'Errigo, 2016(57)	Unspecified	Unspecified	MAC	Unspecified
Cappellari, 2020(58)	Unspecified	Unspecified	CS	Unspecified
Ben-Dor, 2012(59)	Unspecified	Endotracheal intubation	MAC	Propofol, dexmedetomidine ketamine and fentanyl
Althoff, 2021(60)	Unspecified	Unspecified	MAC	Unspecified
Yamamoto, 2013(61)	TIVA	Unspecified	CS	Propofol and remifentanil
HoefUnspecifiedgel, 2023(62)	Unspecified	Unspecified	MAC	Unspecified
Reda, 2012(63)	CIIA	Unspecified	MS	Midazolam
Neumann, 2020(64)	Unspecified	Unspecified	CS	Unspecified

Skutecki, 2022(65)	Unspecified	Unspecified	CS	Unspecified
Liang, 2021(66)	CIIA	Unspecified	CS	Propofol, dexmedetomidine and midazolam and fentanyl
Valente, 2021(67)	Unspecified	Unspecified	CS	Unspecified
Kanda, 2022(68)	CIIA	Endotracheal intubation	CS	Dexmedetomidine and fentanyl
Holmes, 2022(69)	Unspecified	Unspecified	MAC	Unspecified
Liang, 2022(70)	TIVA	Endotracheal intubation	CS	Propofol and remifentanil
Maurice, 2022(71)	TIVA	Endotracheal intubation	Unspecified	Propofol and remifentanil
Sanders, 2021(72)	CIIA	Endotracheal intubation	DS	Propofol, dexmedetomidine and remifentanil
MoUnspecifiedco, 2022(73)	CIIA	Endotracheal intubation	MAC	Midazolam and remifentanil
Goren, 2015(74)	CIIA	Endotracheal intubation	Unspecified	Propofol, etomidate and midazolam
Aslan, 2021(75)	CIIA	Endotracheal intubation	CS	Propofol, midazolam and fentanyl

TIVA: Total intravenous anesthesia; CIIA: Combined intravenous-inhalation anesthesia; CS: Conscious sedation; MS: Moderate sedation; DS: Deep sedation; MAC: Monitored anesthesia care. Unspecified refers to items not described in detail in the study.
