# Supplementary material for: Sedation versus general anesthesia on all-cause mortality in patients undergoing percutaneous procedures: a systematic review and meta-analysis
Source: BMC Anesthesiol. 2024 Apr 2;24:126. doi: 10.1186/s12871-024-02505-w (PMC10985877; doi:10.1186/s12871-024-02505-w)
Supplement: Supplementary file 4 — Supplementary Material 4. [file 12871_2024_2505_MOESM4_ESM.docx]

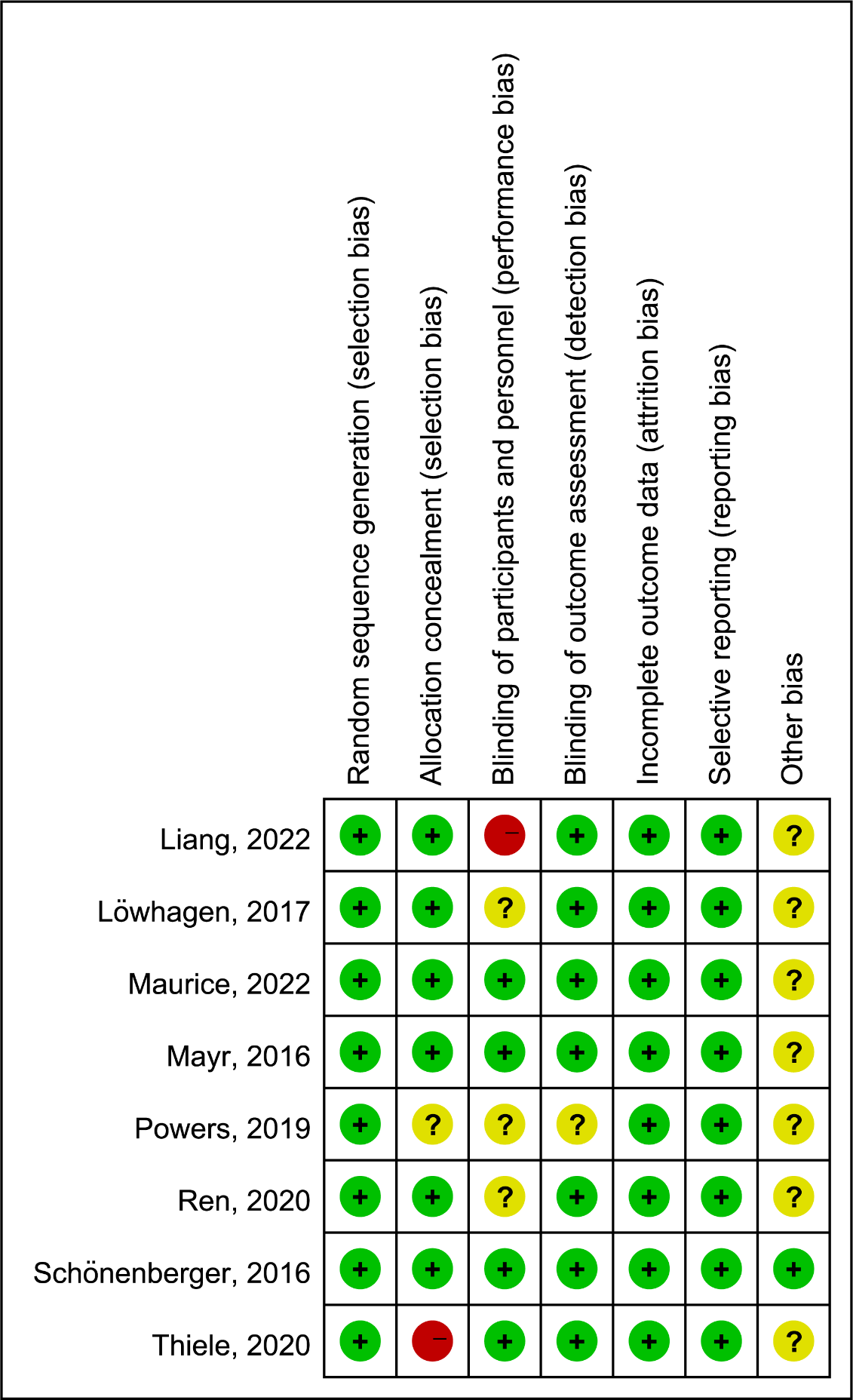


**Supplementary Figure 1** Risk of bias summary for RCTs (randomized controlled trials).


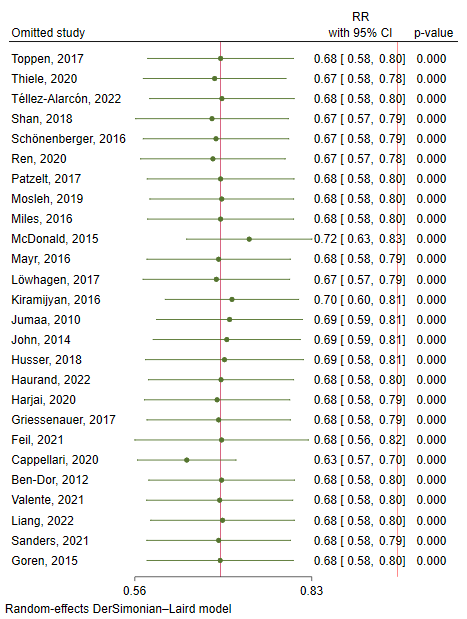


**Supplementary Figure 2** Sensitivity results of sedation versus GA on in-hospital mortality.


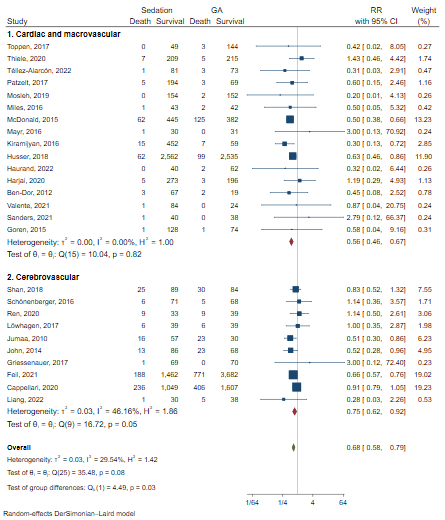


**Supplementary Figure 3** Forest plot illustrating subgroup analysis of pooling in-hospital mortality for type of surgery (cardiac and macrovascular: RR=0.56, 95%CI: 0.46 to 0.67; cerebrovascular: RR=0.75, 95%CI: 0.62 to 0.92).


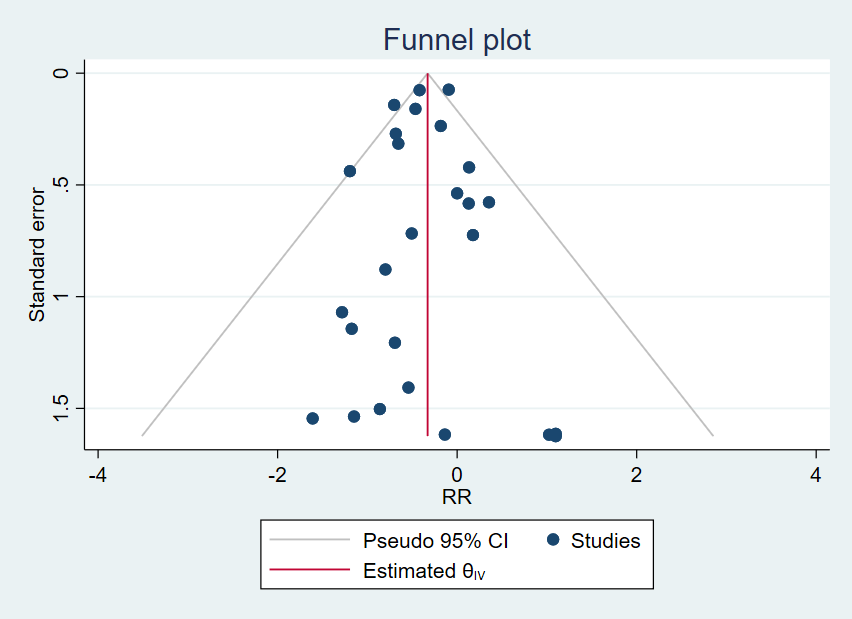


**Supplementary Figure 4** The funnel plot of publication bias of included papers for the comparison on in-hospital mortality.


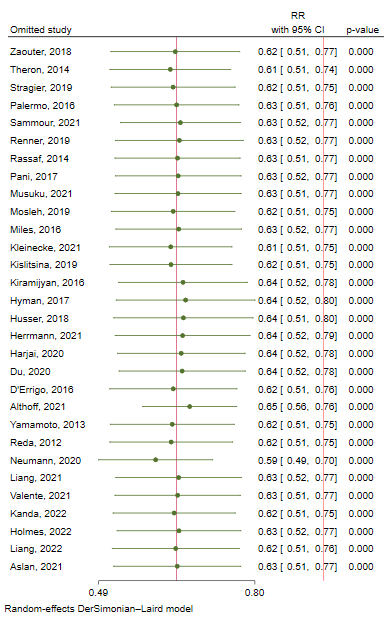


**Supplementary Figure 5** Sensitivity results of sedation versus GA on 30-day mortality.


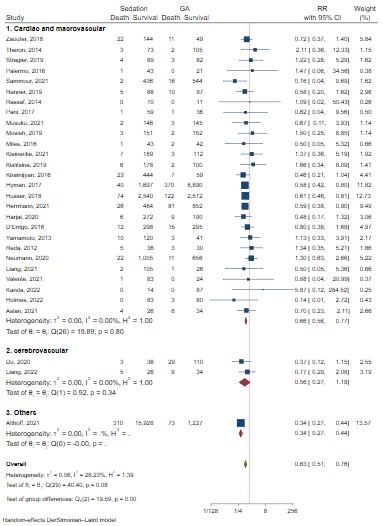


**Supplementary Figure 6** Forest plot illustrating subgroup analysis of pooling 30-day mortality for type of surgery (cardiac and macrovascular: RR=0.66, 95%CI: 0.56 to 0.77; cerebrovascular: RR=0.56, 95%CI: 0.27 to 1.18).


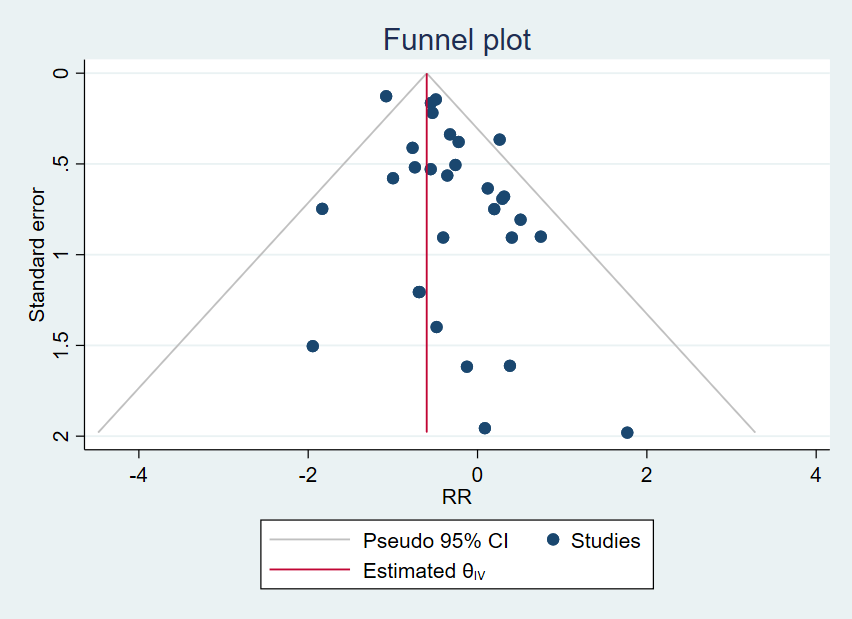


**Supplementary Figure 7** The funnel plot of publication bias of included papers for the comparison on 30-day mortality.


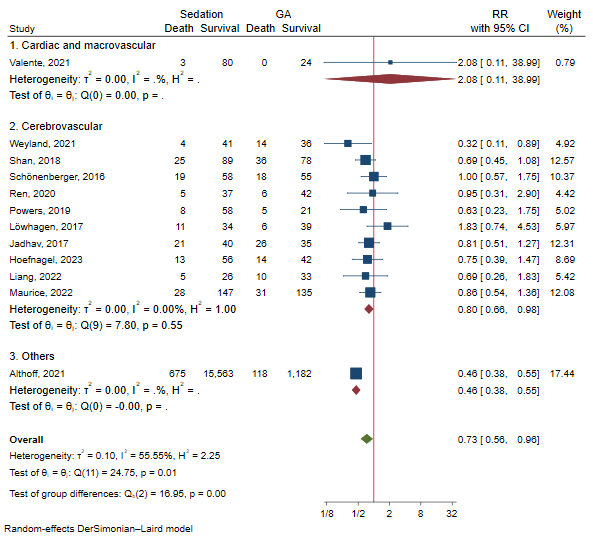


**Supplementary Figure 8** Forest plot illustrating subgroup analysis of pooling 90-day mortality for type of surgery (cerebrovascular: RR=0.80, 95%CI: 0.66 to 0.98).


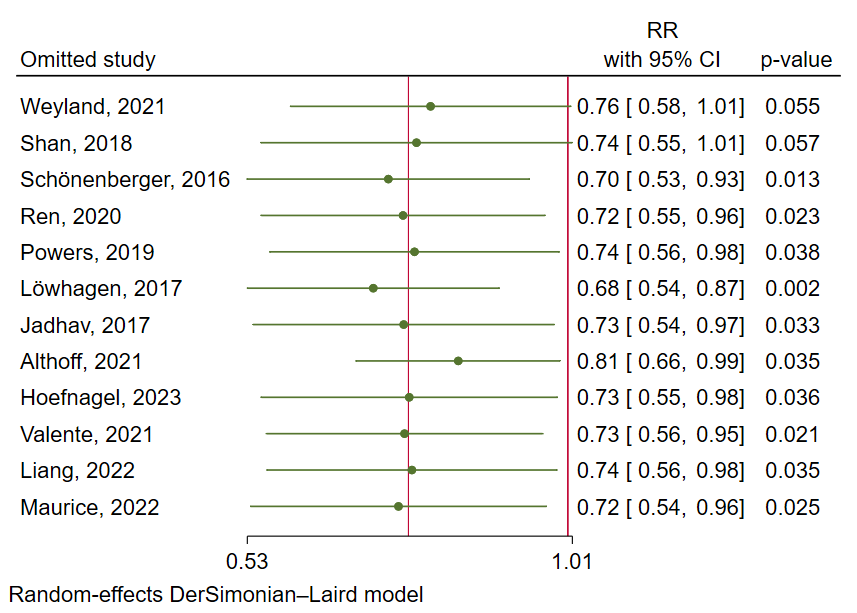


**Supplementary Figure 9** Sensitivity results of sedation versus GA on 90-day mortality.


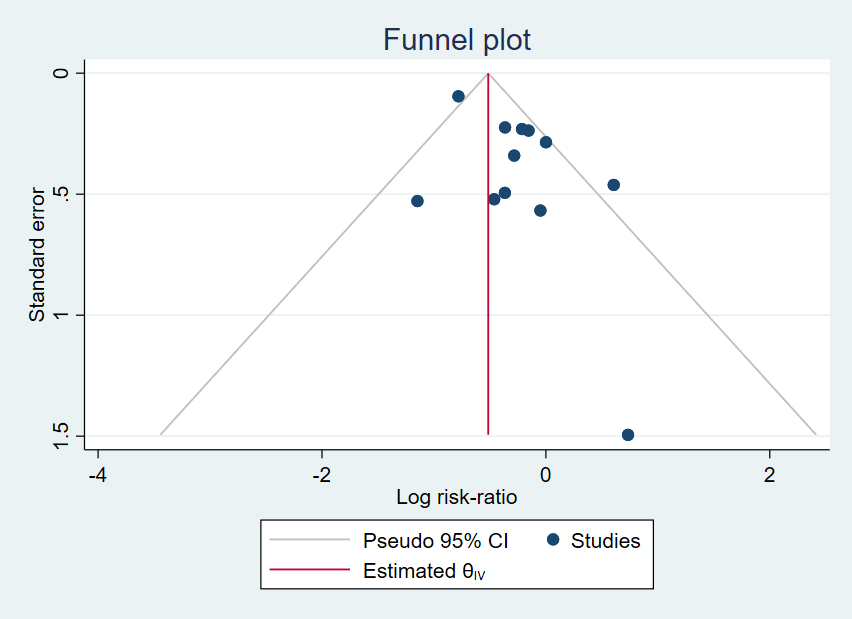


**Supplementary Figure 10** The funnel plot of publication bias of included papers for the comparison on 90-day mortality.
